# Genomic characteristics and nosocomial transmission patterns of different capsular serotypes of *Klebsiella pneumoniae* sequence type 11 in a Chinese hospital

**DOI:** 10.1128/spectrum.02173-26

**Published:** 2026-09-21

**Authors:** Donghong Yang, Han Jia, Ying Shang, Jie Yang, Lili Zhao, Pengcheng Du, Ran Li, Yi Wang

**Affiliations:** 1 Department of Respiratory and Critical Care Medicine, Peking University Peking University People’s Hospital 572420, Beijing, China; 2 Medical Research Center, Beijing Institute of Respiratory Medicine and Beijing Chao-Yang Hospital, Capital Medical University 12517, Beijing, China; 3 Beijing Key Laboratory of Multimodal Intelligent Diagnosis and Treatment System for Respiratory Diseases, Beijing, China; 4 Department of Clinical Laboratory, Beijing Bo’ai Hospital, China Rehabilitation Research Center, Capital Medical University 12517, Beijing, China; Sacramento County Public Health Laboratory, Sacramento, California, USA

**Keywords:** *Klebsiella pneumoniae*, sequence type 11, capsular serotype, carbapenem resistance, virulence gene, nosocomial transmission

## Abstract

Carbapenem-resistant *Klebsiella pneumoniae*, particularly sequence type 11 (ST11), has become the dominant epidemic clone in Asia. In this study, we collected 113 clinical ST11 isolates from a tertiary hospital in Beijing, China. Whole genome sequencing was performed, followed by capsular serotyping, resistome, and virulome analysis, and core-genome single nucleotide polymorphism-based phylogenetic and nosocomial transmission analysis. The aim was to elucidate the distribution of antimicrobial resistance genes, hypervirulence-associated genes, plasmid replicons, and phylogenetic relationships across different capsular serotypes within ST11 *K. pneumoniae*. Associations between the carriages of resistance and virulence genes and plasmid types were also investigated. The dominant capsular serotypes were KL64 (43, 38.1%), KL47 (37, 33.6%), and KL25 (29, 25.7%). The carbapenem resistance gene *bla*_KPC-2_ was nearly ubiquitous (93.8%) across all serotypes, while extended-spectrum β-lactamase genes showed serotype-specific patterns. The carriage of virulence genes differed markedly among serotypes and was linked to distinct plasmid profiles: KL64 isolates frequently harbored *iucA* (83.7%) and *peg-344* (67.4%), whereas KL47 showed a higher prevalence of *rmpA* and *rmpA2*, and KL25 rarely carried virulence genes. These virulence gene profiles were strongly associated with specific plasmid replicons, particularly IncFIB, IncHI1B, and IncR. Phylogenetic analysis revealed clear clustering by serotype, with varying intra-serotype diversity. We identified 20 potential nosocomial transmission events; KL25 was responsible for the majority of within-ward transmissions, while the Respiratory Medicine ward served as a central hub for both intra- and inter-ward spread across serotypes. Our findings reveal substantial genomic and epidemiological heterogeneity among ST11 capsular serotypes.

**IMPORTANCE:**

Capsular polysaccharide (K antigen) serotypes are critical virulence determinants that influence clonal evolution and the emergence of carbapenem-resistant hypervirulent *Klebsiella pneumoniae*. The convergence of resistance and virulence determinants on mobile genetic elements represents a major evolutionary advantage for sequence type 11 (ST11) strains. In this study, our analyses of the distribution of antimicrobial resistance genes, hypervirulence-associated genes, plasmid replicons, and their associations across different capsular serotypes further highlighted the genomic diversity and potential molecular mechanisms associated with the different patterns of resistance and virulence genes across these serotypes. Our findings provide further evidence and suggest that different capsular serotypes may represent distinct evolutionary trajectories within the ST11 lineage. These serotypes also exhibited different patterns during transmission. The acquisition of diverse plasmids might drive the convergence of resistance and virulence and shape serotype-specific transmission dynamics. These results underscore the necessity of integrated genomic surveillance to monitor high-risk clones in healthcare settings.

## INTRODUCTION

*Klebsiella pneumoniae* is a major opportunistic pathogen responsible for a wide spectrum of healthcare-associated infections (HAIs), including pneumonia, bloodstream infections, and urinary tract infections. The rapid global dissemination of carbapenem-resistant *K. pneumoniae* (CRKP) has posed a critical threat to public health, particularly in Asia (1, 2). In China, sequence type 11 (ST11), a single-locus variant of ST258, represents the predominant clone driving the CRKP epidemic, mainly due to the widespread dissemination of *bla*_KPC-2_ (3, 4).

In parallel with the expansion of antimicrobial resistance, increasing evidence highlights the emergence of carbapenem-resistant hypervirulent *K. pneumoniae* (CR-hvKP), especially within the ST11 lineage (5). This convergence of resistance and virulence is frequently mediated by plasmids carrying both resistance determinants and virulence-associated genes, such as *iucA*, *iroB*, *rmpA*, and *peg-344*, leading to strains with enhanced pathogenic potential and limited treatment options (6).

Capsular polysaccharide (K antigen) is a key virulence determinant of *K. pneumoniae* and is also closely associated with clonal evolution and epidemiological success. Among ST11 strains, several capsular serotypes, particularly KL64 and KL47, have been increasingly reported in China and are often linked to CR-hvKP outbreaks (7, 8). Notably, recent genomic studies have suggested that different capsular types within the same sequence type may exhibit distinct evolutionary trajectories, genomic diversity, and differential acquisition of resistance and virulence elements (9, 10).

Plasmids play a central role in shaping the genomic architecture of ST11 *K. pneumoniae*, facilitating the horizontal transfer of resistance and virulence genes. IncFII, IncFIB, IncR, and IncHI1B plasmids have been widely implicated in the dissemination of *bla*_KPC-2_ and virulence-associated loci, contributing to the rapid adaptation and persistence of ST11 clones in hospital settings (11, 12). However, the distribution patterns of plasmid replicons and their association with specific resistance and virulence genes may vary among different capsular serotypes, a topic that remains insufficiently characterized. Furthermore, how this genomic heterogeneity is related to differential nosocomial transmission dynamics has not been well explored.

In this study, we aim to characterize the genomic heterogeneity, evolutionary mechanisms, and transmission patterns underlying different serotypes within the ST11 lineage. We focus on the distribution of antimicrobial resistance genes, hypervirulence-associated genes, and plasmid replicon types among these serotypes. We performed whole genome sequencing (WGS) on ST11 *K. pneumoniae* isolates collected from a tertiary hospital in Beijing, China, systematically compared their genomic characteristics, and explored the potential nosocomial transmissions of the major serotypes.

## MATERIALS AND METHODS

### Isolate collection and identification

Clinical isolates were collected from patients diagnosed with healthcare-associated infections (HAIs) in Beijing Bo’ai Hospital, a Class A tertiary hospital in Beijing, China, between June 2019 and August 2020. All available *K. pneumoniae* isolates obtained during the study period were collected without any pre-selection. The isolates were cultured on Columbia blood agar plates, and monoclonal colonies were selected for taxonomic identification and antimicrobial susceptibility tests using the Vitek compact 2 system.

### Whole genome sequencing and molecular typing

All isolates classified as *K. pneumoniae* by the Vitek compact 2 system were subjected to next-generation sequencing on the NovaSeq 6000 platform (Illumina, San Diego, CA, USA). We used fastp software to obtain the clean data, and assembled the clean reads with SPAdes v4.2.0 (13). Kleborate v3.2.4 was used to confirm taxonomic classification and to identify sequence types, K, and O serotypes based on genome sequences (14). Core genome multilocus sequence typing (cgMLST) was identified using MiST 1.0.0 (15) based on the cgMLST scheme for *K. pneumoniae* (https://bigsdb.pasteur.fr/klebsiella/).

### Genome annotation

Primary gene prediction and annotation were conducted using Prokka v1.14 (16). Antimicrobial resistance genes and virulence genes were identified based on comparisons with ResFinder (17) and Virulence Factor Database (18) by BLAST v2.2.17 (19). PlasmidFinder was used for the *in silico* detection of plasmid replicon sequences (20). The isolates carrying at least one of the genes *iroB*, *iucA*, *peg-344*, *rmpA*, and *rmpA2* were defined as isolates carrying virulence gene(s) (21–23).

### Publicly available data collection and phylogenetic analysis

To explore the phylogenetic relationship of ST11, the chromosomal sequence of *K. pneumoniae* HS11286 (accession no.: NC_016845), which belonged to ST11, was used as the reference for phylogenetic analysis as previously described. Briefly, we compared the assembled genome sequences to the reference and identified the core genome single nucleotide polymorphisms (cgSNPs) using MUMmer v3.23 (24). Recombination sites were detected by Gubbins (25), and the concatenated sequences of filtered polymorphic sites conserved in all genomes were then used for phylogenetic analysis with IQ-tree v2.1.2 (26). The resulting tree was visualized and annotated using iTol (https://itol.embl.de/). Minimum spanning trees based on cgSNPs of the isolates belonging to different serotypes were generated using PHYLOViZ v2.0 (https://github.com/phyloviz).

### Statistical analysis

Statistical analysis and figure generation were conducted in R. The numbers of SNPs among different serotypes were compared by Kruskal-Wallis tests using the kruskal.test() function from the “stats” package, followed by post-hoc Dunn tests using the dunnTest() function in the “FSA” package. The Bonferroni method was used to correct for multiple comparisons. Associations between the carriage of resistance and virulence genes and the plasmid types, and between resistance phenotypes and genotypes were calculated using the fisher.test() function from the “stats” package. The heat map was generated using the “ggplot2” package, and the chordal diagram was generated using the “circlize” package.

## RESULTS

### Clinical characteristics of *K. pneumoniae* ST11 isolates

**Fig 1:**
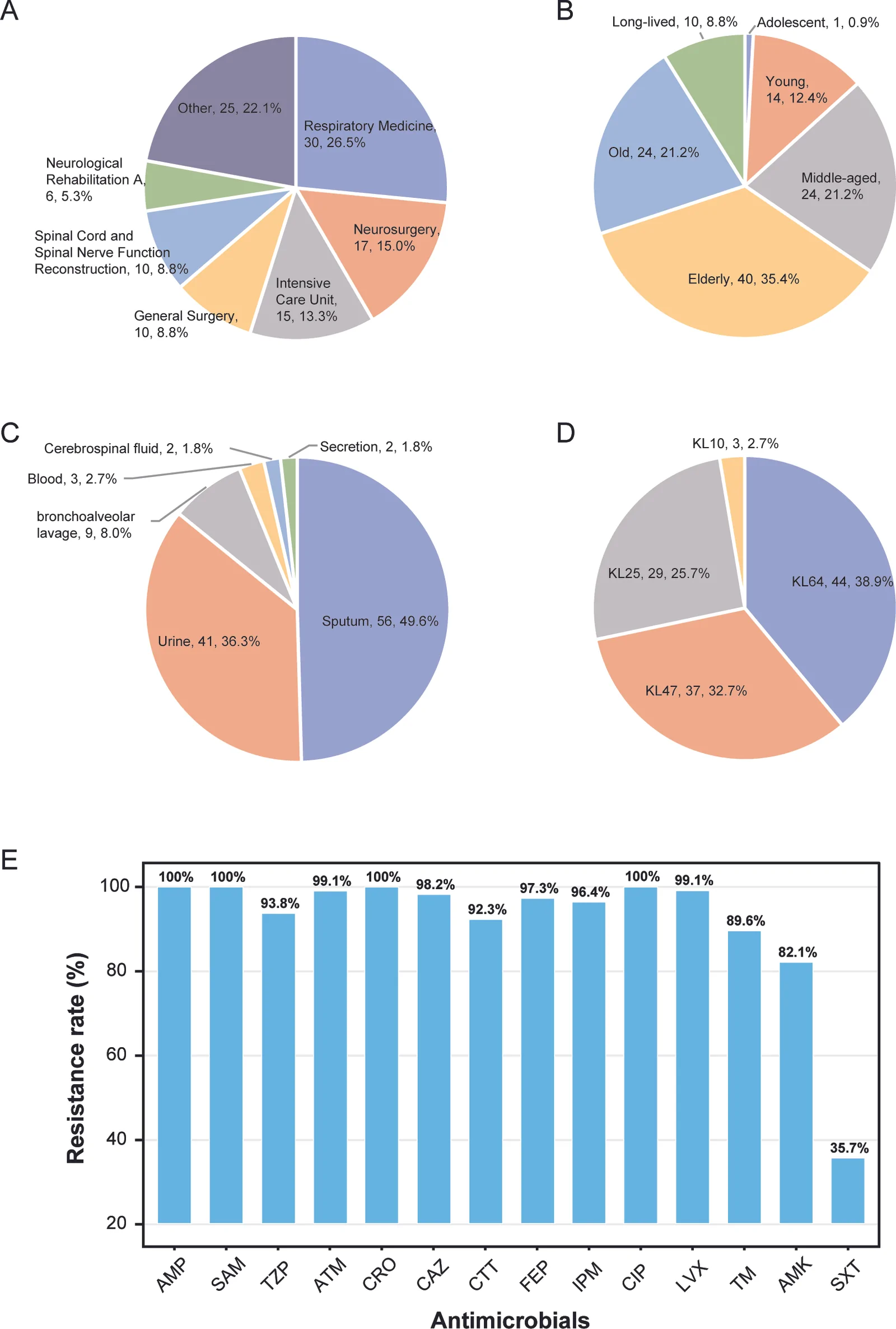
Distribution of isolates by ward (**A**), patient age group (**B**), specimen type (**C**), and capsular serotype (**D**). Age groups are defined as adolescent (<18 years), young (18–44), middle-aged (45–59), elderly (60–74), old (75–89), and long-lived (≥90). (**E**) Antimicrobial resistance rates of the isolates to penicillins (ampicillin [AMP], ampicillin-sulbactam [SAM], and piperacillin-tazobactam [TZP]), monobactams (aztreonam [ATM]), third-generation cephalosporins (ceftriaxone [CRO], ceftazidime [CAZ], and cefotetan [CTT]), fourth-generation cephalosporins (cefepime [FEP]), carbapenems (imipenem [IPM]), fluoroquinolones (ciprofloxacin [CIP] and levofloxacin [LVX]), aminoglycosides (tobramycin [TM] and amikacin [AMK]), and sulfamethoxazole (SXT).

From June 2019 to August 2021, we retrospectively enrolled a total of 94 patients who had at least one clinical culture positive for *K. pneumoniae* during the study period and had isolate(s) available for genomic sequencing and analysis. All these patients were infected during hospitalization and diagnosed with HAIs. The isolates were collected from 18 wards, with the majority from five wards (Fig. 1A): Respiratory Medicine (30 isolates, 26.5%), Neurosurgery (17, 15.0%), General Surgery (10, 8.8%), Spinal Cord and Spinal Nerve Function Reconstruction (10, 8.8%), and Neurological Rehabilitation A (6, 5.3%). The median patient age was 65 years (IQR: 57–78), and most patients were older than 60 years (Fig. 1B). From these patients, we collected 113 isolates from various body sites. Most isolates were from the respiratory and urinary tracts, including 56 (49.6%) from sputum samples, 9 (8.0%) from bronchoalveolar lavage fluid, and 41 (36.3%) from urine samples (Fig. 1C).

### Molecular and phylogenetic characteristics of the Kp isolates

**Fig 2:**
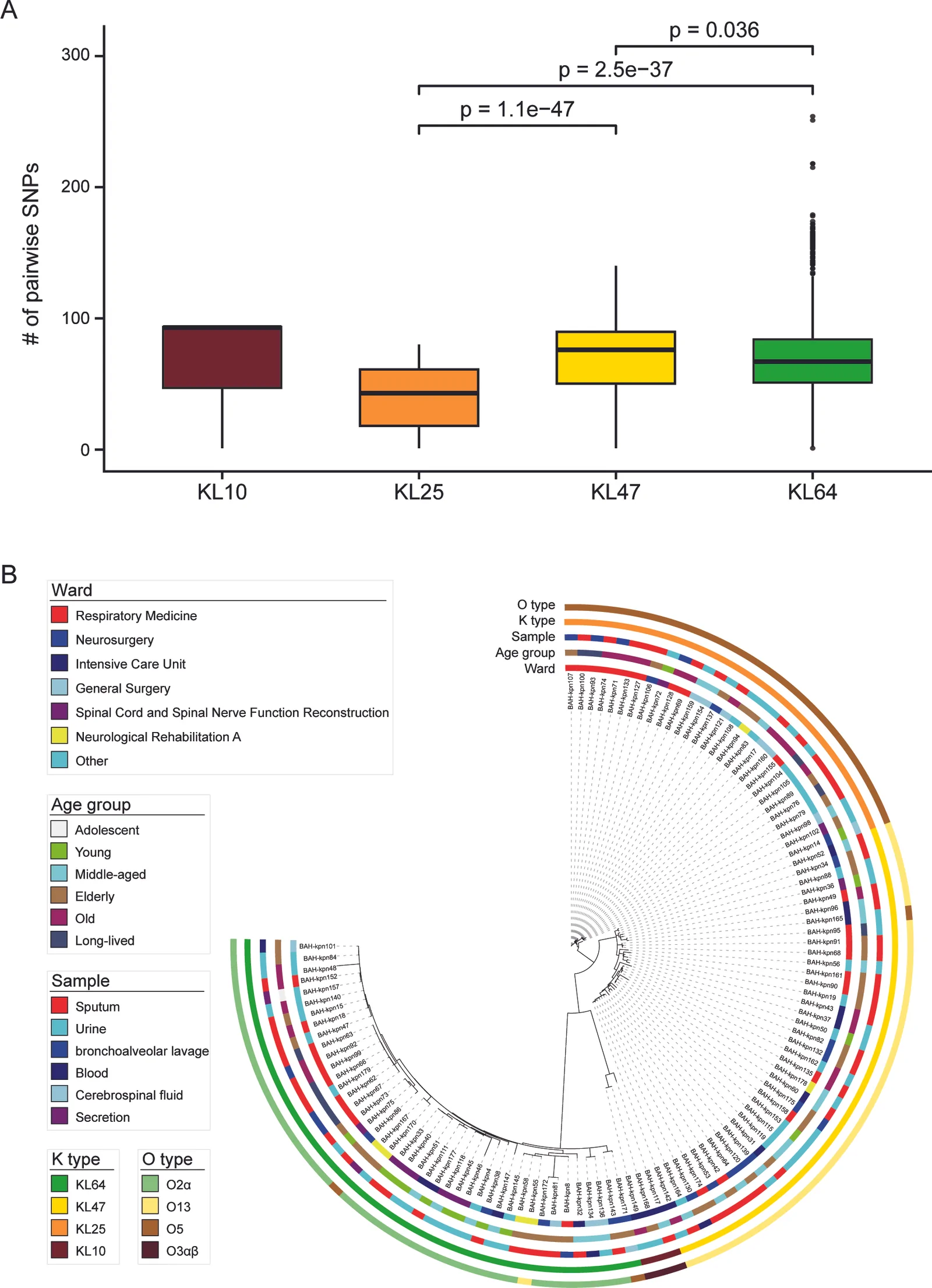
Phylogenetic analysis based on core-genome SNPs (cgSNPs). (**A**) Boxplots showing the pairwise cgSNP differences within each capsular serotype. *P*-values for significant differences between serotypes are annotated. (**B**) Phylogenetic tree based on cgSNPs. The rings, from the inner to outer, represent the wards, patient age groups, specimen types, and capsular serotypes to which each isolate belongs.

All isolates were subjected to WGS. Four K serotypes were identified: KL64 (43 isolates, 38.1%), KL47 (38, 33.6%), KL25 (29, 25.7%), and KL10 (3, 2.7%) (Fig. 1D). Meanwhile, four O serotypes were identified and highly correlated with K serotypes of these isolates, including O2α (all 40 isolates were KL64), O13 (37 KL47 and 1 KL64), O5 (29 KL25, 2 KL64, and 1 KL47), and O3αβ (3 KL10) (Fig. 2B). The three dominant K serotypes displayed significantly different within-serotype diversities, with 73 ± 41 SNPs among KL64, 70 ± 26 SNPs among KL47, and 39 ± 41 SNPs among KL25 (Fig. 2A, *P* < 0.05 for KL64 vs KL47; *P* < 0.001 for KL25 vs KL47 and KL25 vs KL64). In total, 36 cgMLST types were identified: 16 in KL64, 16 in KL47, 2 in KL25, and 2 in KL10, demonstrating substantial clonal diversity among KL64 and KL47 in the hospital. In contrast, 28 of the 29 KL25 isolates (96.6%) belonged to a single cgMLST type, indicating high clonal dissemination. Based on the cgSNPs, a phylogenetic tree was constructed, and four lineages were identified, which aligned perfectly with the K serotype and cgMLST classifications (Fig. 2B).

### Carriage of important antimicrobial resistance genes and mutations among different serotypes

**Fig 3:**
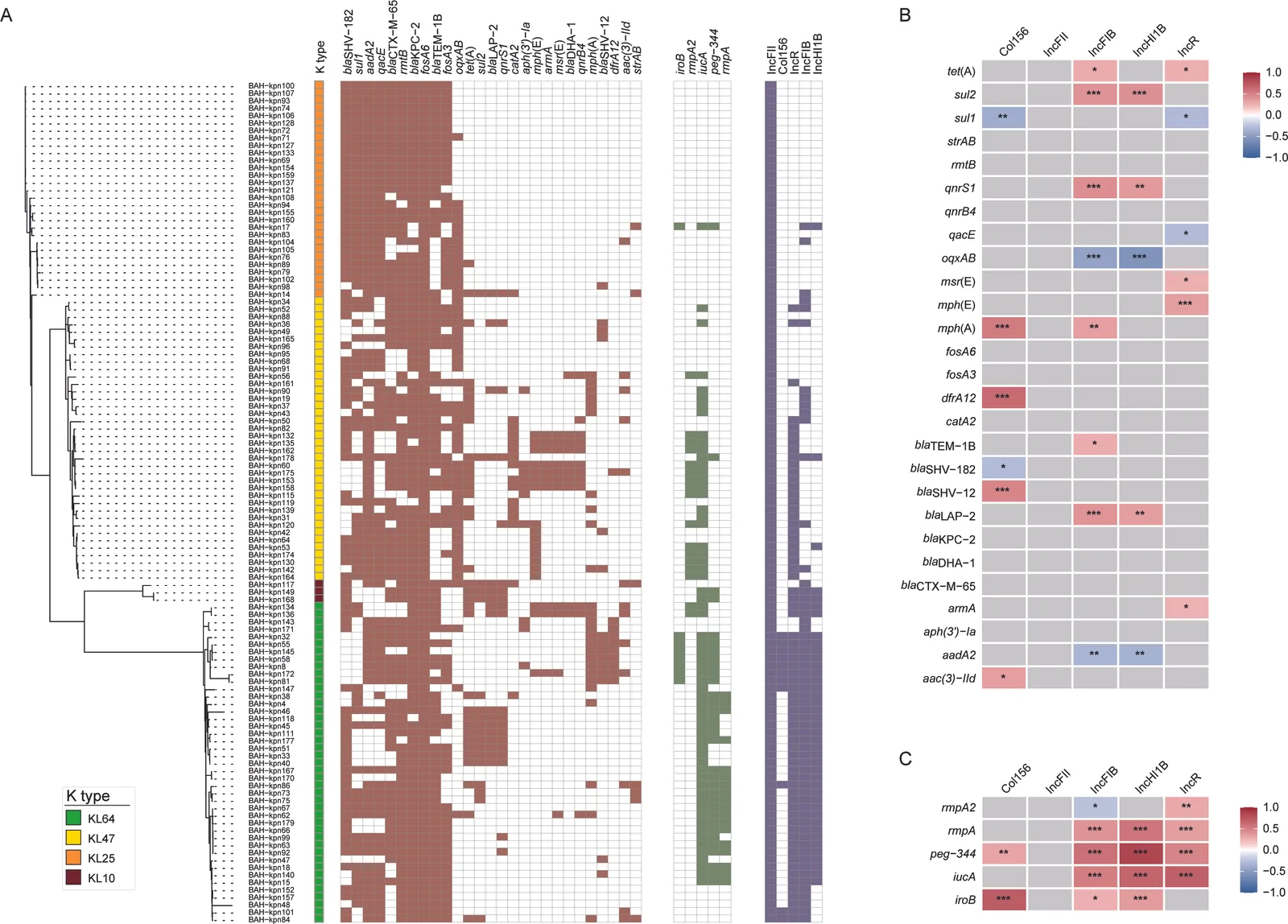
Carriages of resistance genes, virulence genes, and plasmid types, and their associations among the isolates. (**A**) The phylogenetic tree on the left corresponds to that in Fig. 2B. Heatmaps on the right represent the capsular serotypes, and the presence (colored) or absence (white) of resistance genes (red), virulence genes (green), and plasmid types (blue). (**B**) Association between resistance gene carriage and plasmid types. (**C**) Association between virulence gene carriage and plasmid types. Heatmap colors represent the coefficients of association, and the asterisks denote significance levels (*: false discovery rate [FDR] < 0.05, **: FDR < 0.01, ***: FDR < 0.001).

The isolates carried multiple resistance genes; those conferring resistances to carbapenems, beta-lactams, sulfonamides, aminoglycosides, and fosfomycin were the most common (Fig. 3A). For carbapenem resistance, 106 (93.8%) isolates harbored *bla*_KPC-2_ (Table 1), including 42 KL64 isolates (carriage rate: 97.7%), 35 KL47 isolates (94.6%), 26 KL25 isolates (89.7%), and 3 KL10 isolates (100%), demonstrating a high proportion of CRKP among ST11 and across all serotypes. Three families of extended-spectrum beta-lactamase (ESBL) genes were identified: SHV, TEM, and CTX-M types. The most prevalent was the SHV type, carried by 100 (88.5%) isolates, including *bla*_SHV-182_ in 85 (75.2%) and *bla*_SHV-12_ in 15 (13.3%). *Bla*_SHV-182_ was common across the four serotypes, whereas *bla*_SHV-12_ was carried by some of KL64 and KL47 isolates but was rare in KL25 and KL10. The TEM-type ESBL gene *bla*_TEM-1B_ and CTX-M-type ESBL gene *bla*_CTX-M-65_ were also commonly carried by the four serotypes. All isolates were negative for genes conferring colistin and tigecycline resistance. Additionally, two amino acid substitutions in GyrA (S83I and D87G) and one in ParC (S80I) were identified in all isolates, and *qnrB4* was carried by 3 (6.8%) KL64 and 9 (24.3%) KL47 isolates, conferring quinolone resistance. The carriage of these resistance genes and point mutations was in line with the phenotypic resistance profiles. The isolates displayed high resistance rates to the tested antimicrobials, including penicillins, monobactams, third- and fourth-generation cephalosporins, carbapenems, fluoroquinolones, and aminoglycosides (Fig. 1E). *Bla*_KPC-2_ was significantly associated with resistance to imipenem (adjusted *P*: 1.93e−2), piperacillin-tazobactam (adjusted *P*: 7.74e−3), and cefotetan (adjusted *P*: 1.69e−2). *RmtB* was significantly associated with resistance to tobramycin (adjusted *P*: 1.28e−4) and amikacin (adjusted *P*: 3.78e−5). *DfrA12* and *sul2* were significantly associated with resistance to sulfamethoxazole (adjusted *P*: 1.39e−2 and 2.50e−2, respectively).

**TABLE 1:** Carriage of the *bla*_KPC-2_ and ESBL genes among different serotypes

Serotypes	# of isolates	*bla*_KPC-2_	*bla*_SHV-182_	*bla*_SHV-12_	*bla*_TEM-1B_	*bla*_CTX-M-65_
KL64	43	42 (97.7%)^*a*^	30 (69.8%)	9 (20.9%)	38 (88.4%)	28 (65.1%)
KL47	38	35 (92.1%)	24 (63.2%)	5 (13.2%)	23 (60.5%)	28 (73.7%)
KL25	29	26 (89.7%)	28 (96.6%)	1 (3.4%)	21 (72.4%)	25 (86.2%)
KL10	3	3 (100%)	3 (100%)	0 (0%)	3 (100%)	2 (66.7%)
Total	113	106 (93.8%)	85 (75.2%)	15 (13.3%)	85 (75.2%)	83 (73.5%)

^*a*^ Number of isolates carrying the gene and carriage rate among the serotypes.

### Carriage of hypervirulence-associated genes among different serotypes

The carriage of the five hypervirulence associated genes displayed distinct patterns among serotypes (Fig. 3A, Table 2). In total, 63 (55.8%) isolates carried at least one of the five virulence genes: 36 KL64 isolates (positive rate: 81.8%), 24 KL47 isolates (64.9%), 1 KL25 isolate (3.4%), and 2 KL10 isolates (66.7%). The single KL25 and two KL10 isolates carried *iucA* and *peg-344*. Although isolates carrying virulence genes were dominant among the two prevalent serotypes KL64 and KL47, the carriage rates of individual virulence genes differed significantly (Table 2). Among KL64 isolates, the carriage rates were 81.8% for *iucA*, 65.9% for *peg-344*, 15.9% for *iroB*, 4.5% for *rmpA2*, and 0% for *rmpA*. For KL47 isolates, the rates were 64.9% for *iucA*, 2.7% for *peg-344*, 0% for *iroB*, 40.5% for *rmpA2,* and 72.0% for *rmpA*. Carriage of *iucA* was high in both serotypes, while the carriage of the other four genes was significantly different.

**TABLE 2:** Carriage of the five virulence genes among different serotypes

Serotypes	# of isolates	*iucA*	*peg-344*	*iroB*	*rmpA2*	*rmpA*
KL64	43	36 (83.7%)^*a*^	29 (67.4%)	7 (16.3%)	2 (4.7%)	0 (0%)
KL47	38	24 (63.2%)	1 (2.6%)	0 (0%)	15 (39.5%)	18 (47.4%)
KL25	29	1 (3.4%)	1 (3.4%)	1 (3.4%)	0 (0%)	0 (0%)
KL10	3	2 (66.7%)	2 (66.7%)	0 (0%)	0 (0%)	0 (0%)
Total	113	63 (55.8%)	33 (29.2%)	8 (7.1%)	17 (15.0%)	18 (15.9%)

^*a*^ Number of isolates carrying the gene and carriage rate among the serotypes.

### Carriage of plasmids and association with resistance and virulence genes

The carriage of different plasmid types also showed distinct patterns among serotypes (Fig. 3A, Table 3). Five plasmid types were identified. The IncFII type was detected in all 113 isolates. Several other plasmid types were common in KL64 isolates, including IncFIB (43 isolates, carriage rate 100%), IncR (41, 95.3%), and IncHI1B (36, 83.7%). Among KL47 isolates, IncR was common (25 isolates, carriage rate 65.8%), whereas IncFIB was less common (11, 28.9%), and IncHI1B was rare (2, 5.3%). In contrast, KL25 isolates harbored fewer plasmid types; only 3 (10.3%) isolates carried IncFIB and 1 (3.4%) carried IncHI1B in addition to IncFII.

**TABLE 3:** Carriage of the plasmid subtypes among different serotypes

Serotypes	# of isolates	IncFII	Col156	IncR	IncFIB	IncHI1B
KL64	43	43 (100%)^*a*^	11 (25.6%)	41 (95.3%)	43 (100%)	36 (83.7%)
KL47	38	38 (100%)	0 (0%)	25 (65.8%)	11 (28.9%)	2 (5.3%)
KL25	29	29 (100%)	0 (0%)	0 (0%)	3 (10.3%)	1 (3.4%)
KL10	3	3 (100%)	0 (0%)	2 (66.7%)	3 (100%)	2 (66.7%)
Total	113	113 (100%)	11 (9.7%)	68 (60.2%)	60 (53.1%)	41 (36.3%)

^*a*^ Number of isolates carrying the subtype and carriage rate among the serotypes.

The carriage of resistance genes was associated with different plasmid types (Fig. 3B). The genes *mph*(A), *dfrA12*, *bla*_SHV-12_, and *aac (3)-IId* were associated with the Col156 type. *Tet*(A), *sul2*, *qnrS1*, *mph*(A), *bla*_TEM-1B_, and *bla*_LAP-2_ were associated with IncFIB. *Sul2*, *qnrS1,* and *bla*_LAP-2_ were also associated with IncHI1B, while *tet*(A), *msr*(E), *mph*(E), and *armA* were associated with IncR. Of the 27 resistance genes, 7 (25.9%) genes were associated with one plasmid type, 5 (18.5%) with two plasmid types, and the remainder were not significantly associated with any specific plasmid type.

The hypervirulence-associated genes were also related to different plasmid types (Fig. 3C). *RmpA*, *peg-344*, and *iucA* were all associated with IncFIB, IncHI1B, and IncR types. Additionally, *rmpA2* was associated with the IncR type, and *peg-344* was also associated with the Col156 type. *IroB* was associated with Col156, IncFIB, and IncHI1B types. These results indicated that virulence genes are likely carried by different types of plasmids and coexist with multiple resistance genes.

### Nosocomial transmission and spread of the dominant serotypes

**Fig 4:**
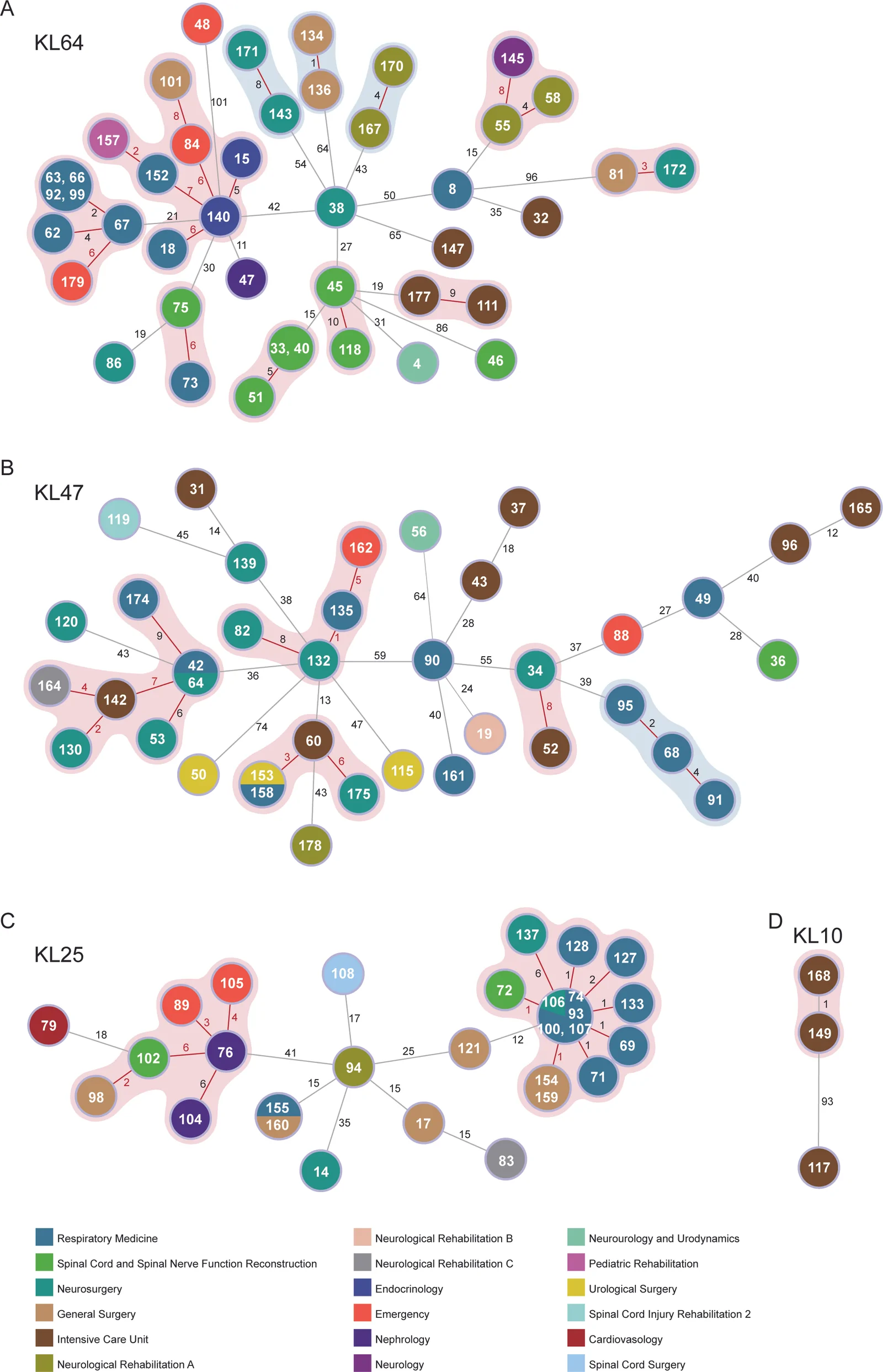
Minimum spanning trees based on cgSNP differences among isolates belonging to KL64 (**A**), KL47 (**B**), KL25 (**C**), and KL10 (**D**). Node labels represent isolate identifiers, and node colors indicate the wards from which the isolates were collected. Edge labels show the number of cgSNPs between two isolates. Edges are colored red if the cgSNP distance is ≤10; labels are red if the two isolates were from different wards. Light red shading highlights clusters comprising isolates from different patients with ≤10 cgSNPs, while light blue shading indicates clusters of multiple isolates from the same patient.

Based on the genomic differences among the isolates, we reconstructed their relationships and identified clusters of isolates with ≤10 pairwise cgSNPs. Among KL64, 11 clusters were identified, encompassing 33 isolates (76.7% of KL64) with 2–7 isolates per cluster (median: 2) (Fig. 4A). Among KL47, five clusters were identified, encompassing 20 isolates (52.6% of KL47) with 2–7 isolates per cluster (median: 4) (Fig. 4B). Among KL25, two clusters were identified, encompassing 20 isolates (69.0% of KL25), with 6 and 14 isolates each (Fig. 4B). These results are consistent with the overall diversity and phylogenetic characteristics of these serotypes (Fig. 2). The K and O serotypes, cgMLST types, and clusters identified based on genomic differences were all not significantly related to the wards where these isolates were obtained.

**Fig 5:**
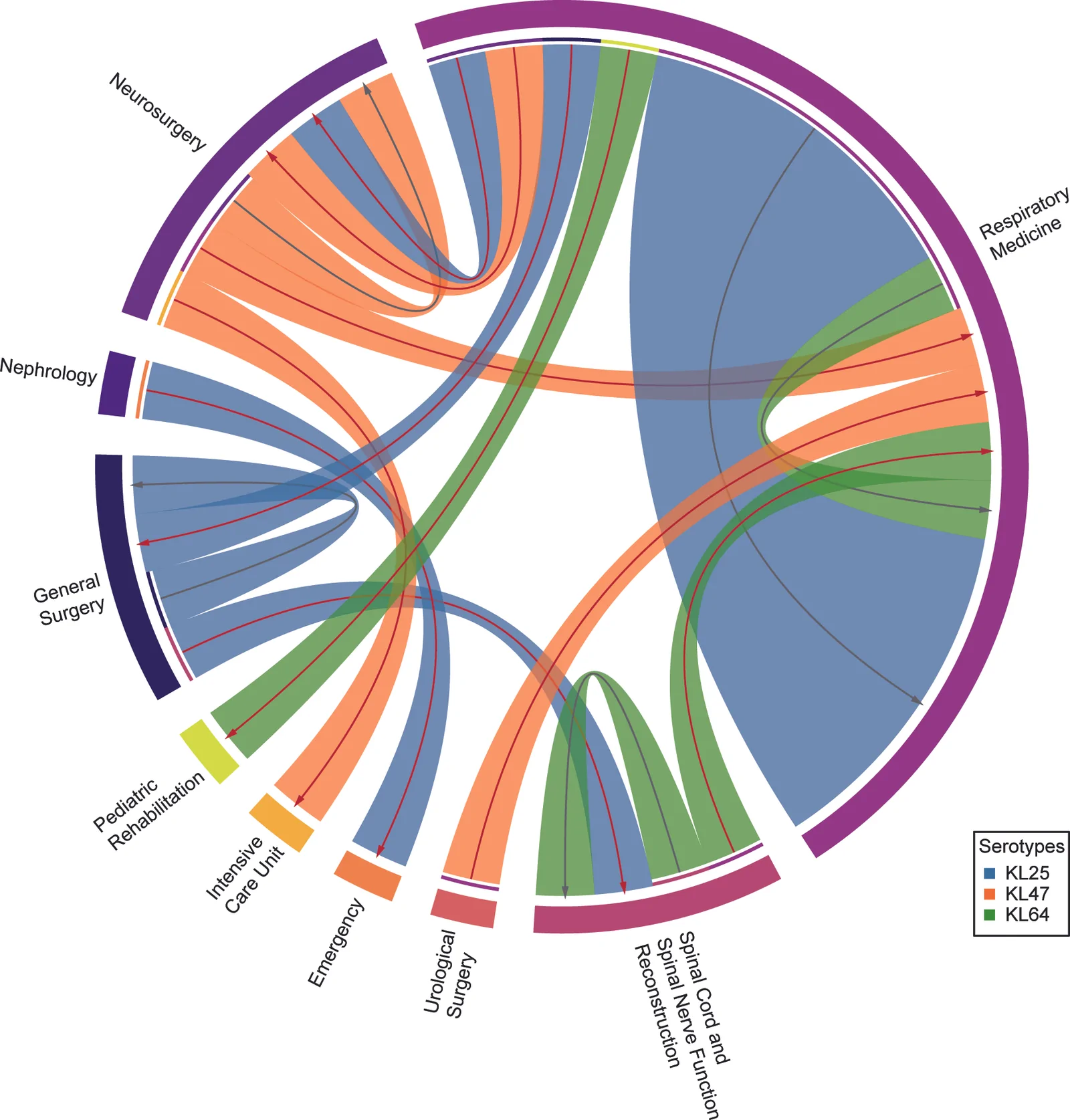
Potential inter- and intra-ward nosocomial transmissions. A potential transmission event between two patients was defined as the recovery of two isolates from different patients within a 30-day window that differed by ≤10 cgSNPs. The outer arcs represent the wards, and the colored ribbons with arrows indicate transmission events. Red arrows denote inter-ward transmissions, and gray arrows denote intra-ward transmissions. Ribbon colors differentiate the serotypes involved in transmission (blue: KL25, orange: KL64, green: KL47).

Within these clusters, we further identified isolate pairs collected from different patients within a 30-day window, which might indicate potential nosocomial transmission. We identified 20 potential transmission events in total: 10 within the same ward (KL25: 7, KL64: 2, KL47: 1) and 10 between two different wards (KL25: 4, KL47: 4, KL64: 2) (Fig. 5). Among the 10 within-ward transmissions, 7 (70.0%) occurred in Respiratory Medicine (KL25: 6, KL64: 1). Among the 10 inter-ward transmissions, 7 (70.0%) were related to Respiratory Medicine, including 4 from Respiratory Medicine to 3 other wards (KL25: 2, KL47: 1, KL64: 1) and 3 from three other wards to Respiratory Medicine (KL47: 2, KL64: 1). These results indicate that Respiratory Medicine was not only the major venue for frequent within-ward transmissions but also the hub for inter-ward transmissions, with multiple serotypes and clones contributing to these events.

## DISCUSSION

In this study, we performed a comprehensive genomic and epidemiological analysis of ST11 *K. pneumoniae* isolates from a single hospital to dissect the heterogeneity among different capsular serotypes, particularly the distribution of resistance and virulence genes, and linked these genetic elements to specific plasmid backbones. Additionally, we also explored the local nosocomial transmission patterns of different serotypes in the hospital.

First, consistent with previous reports, our data confirmed that ST11 remains the dominant CRKP lineage in China, with an extremely high prevalence of *bla*_KPC-2_ across all serotypes and a highly conserved core resistome (3, 27). The nearly universal carriage of *bla*_KPC-2_ across all serotypes (93.8%) demonstrates that carbapenem resistance is a stable and fundamental feature of this lineage. Additionally, the widespread presence of multiple ESBL genes, particularly *bla*_SHV_, *bla*_TEM-1B_, and *bla*_CTX-M-65_, further highlights its multidrug-resistant nature, aligning the genotypic findings with the high-level phenotypic resistance we observed against nearly all tested antimicrobials (Fig. 1E). This phenotypic-genotypic concordance validates the use of genomic surveillance as a reliable proxy for AMR monitoring (28, 29).

Interestingly, we observed serotype-specific differences in resistance gene distribution. For example, *bla*_SHV-12_ and *qnrB4* were more frequently detected in KL47 than in KL25, indicating that certain resistance determinants may preferentially associate with specific capsular backgrounds. Similar observations have been reported in recent studies suggesting that recombination events at the capsule locus may influence the acquisition of accessory resistance genes (28).

Second, our results revealed marked differences in the carriage of hypervirulence-associated genes among serotypes. KL64 and KL47 are the major reservoirs of virulence genes, whereas KL25 showed minimal carriage of virulence genes. Notably, although *iucA* was commonly present in both KL64 and KL47, other virulence genes displayed distinct patterns: KL64 was enriched for *peg-344* and *iroB*, whereas KL47 showed higher prevalence of *rmpA* and *rmpA2*. These findings suggest that different serotypes may harbor distinct virulence plasmids or virulence gene combinations, which is consistent with previous reports indicating multiple evolutionary pathways toward multiple drug resistance and hypervirulence within ST11 (30, 31). The differential distribution of virulence genes may also have clinical implications. Previous studies have shown that *rmpA*/*rmpA2* are associated with hypermucoviscosity and invasive infections, while *iucA* contributes to enhanced iron acquisition and bacterial fitness (32, 33). Therefore, the distinct virulence gene profiles observed in KL64 and KL47 may reflect differences in pathogenic potential and transmission dynamics.

Third, our plasmid analysis further highlighted the genomic diversity and potential molecular mechanisms associated with the different patterns of resistance and virulence genes across these serotypes. We identified significant associations between plasmid types and both resistance and virulence genes. For example, IncFIB and IncHI1B plasmids were strongly associated with key virulence genes (*iucA*, *rmpA*, *peg-344*), supporting previous findings that hybrid resistance–virulence plasmids play a crucial role in the emergence of CR-hvKP (34, 35). Similarly, resistance genes, such as *tet(A*), *sul2*, and *bla*_TEM-1B_ were linked to specific plasmid types, highlighting the importance of plasmids in shaping the resistome of ST11. While IncFII plasmids were universally present, other plasmid types showed strong serotype specificity. The high plasmid burden in KL64—characterized by the co-occurrence of IncFIB, IncHI1B, and IncR replicons—is strongly associated with the carriage of both key resistance genes (*tet*(A), *sul2*) and the *iucA-peg-344* virulence locus. In contrast, KL25 isolates carried fewer plasmids, which may partly explain their lower prevalence of virulence genes.

The convergence of resistance and virulence determinants on mobile genetic elements represents a major evolutionary advantage for ST11 strains. Recent studies have demonstrated that such convergence is often driven by recombination and plasmid fusion events, leading to the emergence of high-risk clones with enhanced fitness and transmissibility (35). Our findings provide further evidence supporting this model and suggest that different capsular serotypes may represent distinct evolutionary trajectories within the ST11 lineage. Furthermore, based on the genomic differences combined with epidemiological information, we identified 20 potential nosocomial transmission events and explored the spread of these serotypes in different wards of the hospital. These serotypes also exhibited different patterns during the transmission. KL25 displayed lower diversities and was dominant in both within- and inter-ward transmissions. Respiratory medicine seems to be the major venue and hub of nosocomial transmission of *K. pneumoniae*, where infection prevention and control measures should be strengthened.

This study has several limitations. It was conducted in a single center, which may limit the generalizability of the findings. The definition of putative transmission events is based on strict genomic and temporal cut-offs and may miss transmissions involving genetically diverse subpopulations or those with longer incubation periods. Functional validation of resistance and virulence phenotypes was not performed and would be required in further studies. Future studies incorporating multicenter sampling and hybrid sequencing approaches are warranted.

### Conclusion

Our study demonstrates that within the dominant ST11 CRKP lineage, capsular serotypes represent fundamentally distinct subclones with significant genomic heterogeneity, divergent virulence gene acquisition strategies, and unique plasmid profiles. While carbapenem resistance is highly conserved across serotypes, the distribution of virulence genes and plasmid types varies markedly. KL64 and KL47 are dominant serotypes linked to high virulence potential and complex plasmid repertoires, serving as crucibles for the evolution of CR-hvKP. Meanwhile, KL25, though less virulent, is a highly efficient epidemic clone driving local outbreaks. The differential association of resistance and virulence genes with specific plasmid types highlights the critical role of mobile genetic elements in the evolution of ST11. These findings provide important insights into the genomic basis of serotype-specific characteristics and underscore the need for continuous genomic surveillance to monitor the emergence of high-risk clones.

## Data Availability

The sequencing data generated in this study have been deposited in the Genome Sequence Archive in National Genomics Data Center, China National Center for Bioinformation/Beijing Institute of Genomics, Chinese Academy of Sciences that are publicly accessible at https://ngdc.cncb.ac.cn/bioproject/browse/PRJCA073933.

## References

[1] Wyres KL, Nguyen TNT, Lam MMC, Judd LM, van Vinh Chau N, Dance DAB, Ip M, Karkey A, Ling CL, Miliya T, Newton PN, Lan NPH, Sengduangphachanh A, Turner P, Veeraraghavan B, Vinh PV, Vongsouvath M, Thomson NR, Baker S, Holt KE. 2020. Genomic surveillance for hypervirulence and multi-drug resistance in invasive *Klebsiella pneumoniae* from South and Southeast Asia. Genome Med 12:11. doi:10.1186/s13073-019-0706-y PMC6966826 31948471

[2] Guo H, Wu Y, Li L, Wang J, Xu J, He F. 2023. Global emergence of carbapenem-resistant *Klebsiella pneumoniae* co-carrying multiple carbapenemases. Comput Struct Biotechnol J 21:3557–3563. doi:10.1016/j.csbj.2023.07.013 PMC10368534 37501706

[3] Shi Q, Ruan Z, Zhang P, Hu H, Han X, Wang Z, Lou T, Quan J, Lan W, Weng R, Zhao D, Du X, Yu Y, Jiang Y. 2024. Epidemiology of carbapenem-resistant *Klebsiella pneumoniae* in China and the evolving trends of predominant clone ST11: a multicentre, genome-based study. J Antimicrob Chemother 79:2292–2297. doi:10.1093/jac/dkae227 38997220

[4] Zhou K, Xue C-X, Xu T, Shen P, Wei S, Wyres KL, Lam MMC, Liu J, Lin H, Chen Y, Holt KE, BRICS Working Group, Xiao Y. 2023. A point mutation in recC associated with subclonal replacement of carbapenem-resistant *Klebsiella pneumoniae* ST11 in China. Nat Commun 14:2464. doi:10.1038/s41467-023-38061-z PMC10147710 37117217

[5] Gu D, Dong N, Zheng Z, Lin D, Huang M, Wang L, Chan E-C, Shu L, Yu J, Zhang R, Chen S. 2018. A fatal outbreak of ST11 carbapenem-resistant hypervirulent *Klebsiella pneumoniae* in a Chinese hospital: a molecular epidemiological study. Lancet Infect Dis 18:37–46. doi:10.1016/S1473-3099(17)30489-9 28864030

[6] Li D, Fan Z, Wang S, Su Y, Yuan M, Fang H, Wang R, Gao H, Bai H, Mao D, Luo Y. 2026. Plasmid-mediated convergence of multidrug resistance and hypervirulence in community-acquired *Klebsiella pneumoniae* in Tianjin, China. BMC Microbiol 26:352. doi:10.1186/s12866-026-04867-2 PMC13081646 41794678

[7] Hu F, Pan Y, Li H, Han R, Liu X, Ma R, Wu Y, Lun H, Qin X, Li J, Wang A, Zhou M, Liu B, Zhou Z, He P. 2024. Carbapenem-resistant *Klebsiella pneumoniae* capsular types, antibiotic resistance and virulence factors in China: a longitudinal, multi-centre study. Nat Microbiol 9:814–829. doi:10.1038/s41564-024-01612-1 PMC10914598 38424289

[8] Chen H, Li T, Huang X, He Q. 2025. Emergence of ST11-KL64 carbapenem-resistant hypervirulent *Klebsiella pneumoniae* isolates harboring blaKPC−2 and iucA from a tertiary teaching hospital in Western China. BMC Infect Dis 25:880. doi:10.1186/s12879-025-11241-6 PMC12220110 40596916

[9] Li Y-T, Wang Y-C, Chen C-M, Tang H-L, Chen B-H, Teng R-H, Chiou C-S, Lu M-C, Lai Y-C. 2023. Distinct evolution of ST11 KL64 *Klebsiella pneumoniae* in Taiwan. Front Microbiol 14:1291540. doi:10.3389/fmicb.2023.1291540 PMC10748404 38143864

[10] Chen T, Wang Y, Zhou Y, Zhou W, Chi X, Shen P, Zheng B, Xiao Y. 2023. Recombination drives evolution of carbapenem-resistant *Klebsiella pneumoniae* sequence type 11 KL47 to KL64 in China. Microbiol Spectr 11:e01107-22. doi:10.1128/spectrum.01107-22 PMC9927301 36622219

[11] Zhang F, Li L, Zhao Y, Dong H, Zhao B, Zhao X, Xia Z, Chi L, Wang Y, Li R, Qin S, Fu X. 2024. Molecular characterization of hybrid virulence plasmids in ST11-KL64 KPC-2-producing multidrug-resistant hypervirulent *Klebsiella pneumoniae* from China. Front Microbiol 15:1353849. doi:10.3389/fmicb.2024.1353849 PMC10972857 38550871

[12] Hong H-X, Huo B-H, Xiang T-X, Wei D-D, Huang Q-S, Liu P, Zhang W, Xu Y, Liu Y. 2024. Virulence plasmid with IroBCDN deletion promoted cross-regional transmission of ST11-KL64 carbapenem-resistant hypervirulent *Klebsiella pneumoniae* in central China. BMC Microbiol 24:400. doi:10.1186/s12866-024-03564-2 PMC11465609 39385085

[13] Prjibelski A, Antipov D, Meleshko D, Lapidus A, Korobeynikov A. 2020. Using SPAdes *de novo* assembler. CP in Bioinformatics 70:e102. doi:10.1002/cpbi.102 32559359

[14] Lam MMC, Wick RR, Watts SC, Cerdeira LT, Wyres KL, Holt KE. 2021. A genomic surveillance framework and genotyping tool for *Klebsiella pneumoniae* and its related species complex. Nat Commun 12:4188. doi:10.1038/s41467-021-24448-3 PMC8263825 34234121

[15] Bogaerts B, Roosens NHC, Vanneste K. 2025. MiST: rapid, accurate and flexible (core-genome) multi-locus sequence typing (MLST) allele calling from draft genomes. BMC Genomics 26:1106. doi:10.1186/s12864-025-12324-z PMC12709719 41257567

[16] Seemann T. 2014. Prokka: rapid prokaryotic genome annotation. Bioinformatics 30:2068–2069. doi:10.1093/bioinformatics/btu153 24642063

[17] Florensa AF, Kaas RS, Clausen P, Aytan-Aktug D, Aarestrup FM. 2022. ResFinder – an open online resource for identification of antimicrobial resistance genes in next-generation sequencing data and prediction of phenotypes from genotypes. Microb Genom 8:000748. doi:10.1099/mgen.0.000748 PMC8914360 35072601

[18] Zhou S, Liu B, Zheng D, Chen L, Yang J. 2025. VFDB 2025: an integrated resource for exploring anti-virulence compounds. Nucleic Acids Res 53:D871–D877. doi:10.1093/nar/gkae968 PMC11701737 39470738

[19] Altschul SF, Gish W, Miller W, Myers EW, Lipman DJ. 1990. Basic local alignment search tool. J Mol Biol 215:403–410. doi:10.1016/S0022-2836(05)80360-2 2231712

[20] Carattoli A, Zankari E, García-Fernández A, Voldby Larsen M, Lund O, Villa L, Møller Aarestrup F, Hasman H. 2014. *In silico* detection and typing of plasmids using PlasmidFinder and plasmid multilocus sequence typing. Antimicrob Agents Chemother 58:3895–3903. doi:10.1128/AAC.02412-14 PMC4068535 24777092

[21] Russo TA, Olson R, Fang C-T, Stoesser N, Miller M, MacDonald U, Hutson A, Barker JH, La Hoz RM, Johnson JR. 2018. Identification of biomarkers for differentiation of hypervirulent *Klebsiella pneumoniae* from classical *K. pneumoniae*. J Clin Microbiol 56:e00776-18. doi:10.1128/JCM.00776-18 PMC6113484 29925642

[22] Tang Y, Du P, Du C, Yang P, Shen N, Russo TA, Liu C. 2025. Genomically defined hypervirulent *Klebsiella pneumoniae* contributed to early-onset increased mortality. Nat Commun 16:2096. doi:10.1038/s41467-025-57379-4 PMC11873152 40025046

[23] Russo TA, Lebreton F, McGann PT. 2025. A step forward in hypervirulent Klebsiella pneumoniae diagnostics. Emerg Infect Dis 31:1–3. doi:10.3201/eid3101.241516 PMC11682795 39714290

[24] Kurtz S, Phillippy A, Delcher AL, Smoot M, Shumway M, Antonescu C, Salzberg SL. 2004. Versatile and open software for comparing large genomes. Genome Biol 5:R12. doi:10.1186/gb-2004-5-2-r12 PMC395750 14759262

[25] Croucher NJ, Page AJ, Connor TR, Delaney AJ, Keane JA, Bentley SD, Parkhill J, Harris SR. 2015. Rapid phylogenetic analysis of large samples of recombinant bacterial whole genome sequences using Gubbins. Nucleic Acids Res 43:e15. doi:10.1093/nar/gku1196 PMC4330336 25414349

[26] Minh BQ, Schmidt HA, Chernomor O, Schrempf D, Woodhams MD, von Haeseler A, Lanfear R. 2020. IQ-TREE 2: new models and efficient methods for phylogenetic inference in the genomic era. Mol Biol Evol 37:1530–1534. doi:10.1093/molbev/msaa015 PMC7182206 32011700

[27] Ma Z, Mo L, Hu J, Qu J, Cao H, Jiang S. 2025. Emergence and regional evolution of *Klebsiella pneumoniae* carbapenemase (KPC)-producing *Klebsiella pneumoniae* ST11 in China. BMC Genomics 26:1120. doi:10.1186/s12864-025-12314-1 PMC12751682 41257553

[28] Li P, Xiao Y, Luo R, Zhang J, Nie F, Zou T, Liu Y, Zhang W, Xiang T. 2026. Comparative genomic, phenotypic, and clinical characterization of ST11-KL25 and ST11-KL64 hypervirulent carbapenem-resistant *Klebsiella pneumoniae* in a tertiary hospital, 2020-2023. Antimicrob Agents Chemother 70:e0108025. doi:10.1128/aac.01080-25 PMC13041405 41711572

[29] Karlowsky JA, Lob SH, DeRyke CA, Siddiqui F, Young K, Motyl MR, Sahm DF. 2022. Prevalence of ESBL non-CRE *Escherichia coli* and *Klebsiella pneumoniae* among clinical isolates collected by the SMART global surveillance programme from 2015 to 2019. Int J Antimicrob Agents 59:106535. doi:10.1016/j.ijantimicag.2022.106535 35091052

[30] Wang R, Zhang A, Sun S, Yin G, Wu X, Ding Q, Wang Q, Chen F, Wang S, van Dorp L, Zhang Y, Jin L, Wang X, Balloux F, Wang H. 2024. Increase in antioxidant capacity associated with the successful subclone of hypervirulent carbapenem-resistant *Klebsiella pneumoniae* ST11-KL64. Nat Commun 15:67. doi:10.1038/s41467-023-44351-3 PMC10761919 38167298

[31] Yang Y, Qin J, Hu Y, Wang J, Feng Y, Zong Z. 2026. Carbapenem-resistant *Klebsiella pneumoniae* of sequence type 11: a scoping review. J Infect Dis 233:S72–S80. doi:10.1093/infdis/jiaf635 41824646

[32] Russo TA, Alvarado CL, Davies CJ, Drayer ZJ, Carlino-MacDonald U, Hutson A, Luo TL, Martin MJ, Corey BW, Moser KA, Rasheed JK, Halpin AL, McGann PT, Lebreton F. 2024. Differentiation of hypervirulent and classical *Klebsiella pneumoniae* with acquired drug resistance. mBio 15:e02867-23. doi:10.1128/mbio.02867-23 PMC10865842 38231533

[33] Hernandez GE, Valencia-Bacca JD, Bennett EF, Islam MM, Maiti S, Nutter NA, Young TM, Caudell DL, Costa-Terryll A, Skelly JJ, Floyd ET, Atkins HM, Zafar MA. 2026. Aerobactin is a key driver of hypervirulent *Klebsiella pneumoniae* translocation and virulence. PLoS Pathog 22:e1014122. doi:10.1371/journal.ppat.1014122 PMC13089870 41973764

[34] Xu Q, Sun R, Liu X, Heng H, Yang X, Xie M, Yang C, Ye L, Chan EW-C, Zhang R, Chen S. 2025. Global dissemination of conjugative virulence plasmids co-harboring hypervirulence and multidrug resistance genes in *Klebsiella pneumoniae*. mSystems 10:e01675-24. doi:10.1128/msystems.01675-24 PMC12013265 40130870

[35] Gibbon MJ, Couto N, Cozens K, Habib S, Cowley L, Aanensen DM, Corander J, Thorpe HA, Hetland MAK, Sassera D, Merla C, Corbella M, Ferrari C, Turner KME, Sirikanchana K, Dulyayangkul P, Charoenlap N, Thamlikitkul V, Avison MB, Feil EJ. 2026. Convergence and global molecular epidemiology of *Klebsiella pneumoniae* plasmids harbouring the iuc3 virulence locus: a population genomic analysis. Lancet Microbe 7:101236. doi:10.1016/j.lanmic.2025.101236 41512896

